# Optimal Application of Fractional Flow Reserve to Assess Serial Coronary Artery Disease: A 3D‐Printed Experimental Study With Clinical Validation

**DOI:** 10.1161/JAHA.118.010279

**Published:** 2018-10-14

**Authors:** Bhavik N. Modi, Matthew Ryan, Anjalee Chattersingh, Kseniia Eruslanova, Howard Ellis, Nicholas Gaddum, Jack Lee, Brian Clapp, Phil Chowienczyk, Divaka Perera

**Affiliations:** ^1^ NIHR Biomedical Research Centre and British Heart Foundation Centre of Excellence School of Cardiovascular Medicine and Sciences St Thomas’ Campus King's College London London United Kingdom; ^2^ School of Biomedical Engineering and Imaging Sciences King's College London London United Kingdom

**Keywords:** coronary artery disease, fractional flow reserve, percutaneous coronary intervention, Catheter-Based Coronary and Valvular Interventions, Percutaneous Coronary Intervention, Revascularization, Physiology, Translational Studies

## Abstract

**Background:**

Assessing the physiological significance of stenoses with coexistent serial disease is prone to error. We aimed to use 3‐dimensional‐printing to characterize serial stenosis interplay and to derive and validate a mathematical solution to predict true stenosis significance in serial disease.

**Methods and Results:**

Fifty‐two 3‐dimensional‐printed serial disease phantoms were physiologically assessed by pressure‐wire pullback (ΔFFR_app_) and compared with phantoms with the stenosis in isolation (ΔFFR_true_). Mathematical models to minimize error in predicting FFR_true_, the FFR in the vessel where the stenosis is present in isolation, were subsequently developed using 32 phantoms and validated in another 20 and also a clinical cohort of 30 patients with serial disease. ΔFFR_app_ underestimated ΔFFR_true_ in 88% of phantoms, with underestimation proportional to total FFR. Discrepancy as a proportion of ΔFFR_true_ was 17.1% (absolute difference 0.036±0.048), which improved to 2.9% (0.006±0.023) using our model. In the clinical cohort, discrepancy was 38.5% (0.05±0.04) with 13.3% of stenoses misclassified (using FFR <0.8 threshold). Using mathematical correction, this improved to 15.4% (0.02±0.03), with the proportion of misclassified stenoses falling to 6.7%.

**Conclusions:**

Individual stenoses are considerably underestimated in serial disease, proportional to total FFR. We have shown within in vitro and clinical cohorts that this error is significantly improved using a mathematical correction model, incorporating routinely available pressure‐wire pullback data.

**Clinical Perspective**

**What Is New?**

- Individual stenoses are considerably underestimated in serial disease, proportional to total fractional flow reserve.
- This study provides operators with a simple mathematical correction model to reduce this error that can be readily incorporated into routine daily practice.

**What Are the Clinical Implications?**

- Our findings should make operators wary when attempting to identify the individual physiological significance of stenoses in serially diseased vessels, particularly when the total vessel fractional flow reserve is low.
- We provide operators with a mathematical correction model, incorporating routinely available pressure‐wire pullback data, to minimize the error of performing fractional flow reserve assessment of the physiological significance of individual stenosis.

## Introduction

Growing evidence supports ischemia‐guided revascularization for coronary artery disease (CAD).^1^ Ischemia assessment has now evolved to be able to assess physiologically significant disease in specific vessels, at the time of diagnostic angiography. This involves calculating the ratio of distal coronary pressure to aortic pressure at maximal hyperemia (fractional flow reserve, FFR),^2^ at rest during the entire cardiac cycle (P_d_/P_a_)^3^ or at rest during a defined‐phase of diastole (instantaneous wave‐free ratio, iFR).^4^ Physiology‐guided revascularization appears to confer significant clinical and prognostic benefit over management based on angiographic appearance alone.^5^, ^6^, ^7^

While these pressure‐derived indices are well established and validated for vessels with single lesions, given the systemic nature of atherosclerosis, coronary stenoses are often found in tandem with other focal or diffusely diseased segments within the same vessel. In such instances, the decision to revascularize and the mode of revascularization chosen rely on identifying the true individual contribution of lesions and not just the cumulative impact of disease in the entire vessel. However, each diseased segment affects the fluid dynamics of the other, which makes it difficult to apply conventional physiological techniques to identify the true significance of a lesion within a serially diseased vessel.^8^, ^9^ Knowing the true functional significance of each stenosis enables the appropriate stenosis to be targeted and helps ensure the correct revascularization strategy is chosen, whether this is percutaneous coronary intervention (PCI), coronary artery bypass graft, or medical therapy.

Theoretical solutions have been developed that involve complex formulae^10^ using measurements of intracoronary pressure at various points in the artery and determination of coronary wedge pressure, which in turn is acquired during transient balloon occlusion of the vessel.^8^, ^11^ The rationale for incorporating a wedge pressure measurement was to standardize and account for variability in collateral flow across a stenosis.^11^ A major drawback of this technique, which has limited the adoption of these formulae in clinical practice, is that in order to obtain a wedge pressure measurement, it is necessary to balloon occlude the coronary artery, which carries a risk of dissection. This could have clinical sequelae if angiographically undetectable at the time, or if detected, may mandate stenting regardless of the results of physiological assessment. Other solutions to this problem have relied on the identification of a large disease‐free side branch^12^, ^13^ to separate the true significance of a proximal stenosis, although this scenario is only applicable to left main coronary artery disease where one of the daughter vessels is free of disease. Furthermore, the relative accuracy of hyperemic versus resting indices, such as iFR,^14^ in the context of serial disease is unknown.

The aim of this study was first to develop an in vitro model, based on 3‐dimensional (3D)‐printed configurations of tandem disease, that is a realistic representation of pulsatile coronary circulation in hyperemic conditions. Using these results, we aimed to characterize the factors that influence serial stenosis hemodynamic interplay and incorporate them in a theoretical model to improve prediction of the true FFR of individual stenoses, without the need for wedge pressure measurements or angiographic guesses of stenosis and vessel diameters. We then sought to test such a solution within a clinical cohort of patents with serial CAD.

## Methods

The data, analytic methods, and study materials will not be made available to other researchers for purposes of reproducing the results or replicating the procedure.

### Creating a Model of Tandem Coronary Artery Disease

**Figure 1:**
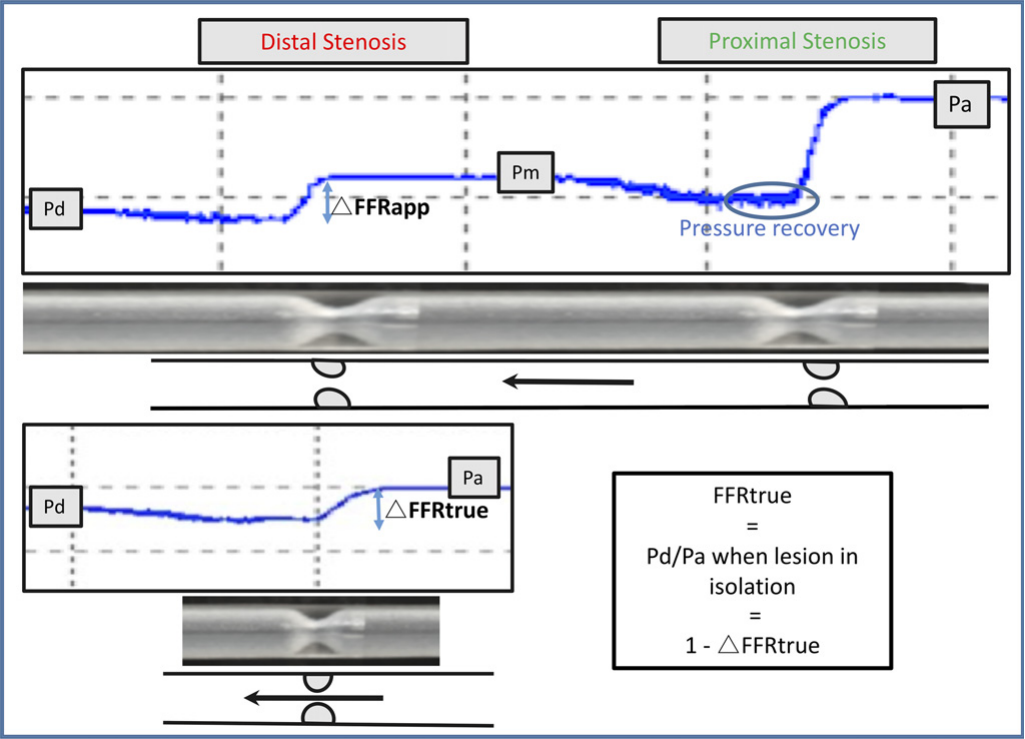
How 3‐dimensional (3D) printing was used to model pressure‐wire measurements in serial disease. *Top:* Photographs of a 3D‐printed tube modeling tandem lesions with a pressure‐wire pullback demonstrating distal coronary pressure (P_d_), pressure between lesions (P_m_), and aortic pressure (P_a_). ∆FFR_app_ represents the apparent pressure gradient across a stenosis when in the presence of another. Also demonstrated is the phenomenon of pressure recovery after a tight stenosis, showing further evidence of our model replicating in vivo physiology. *Bottom:* 3D‐printed tube with the corresponding single stenosis in isolation. ∆FFR_true_ represents the true pressure gradient across a stenosis.

**Figure 2:**
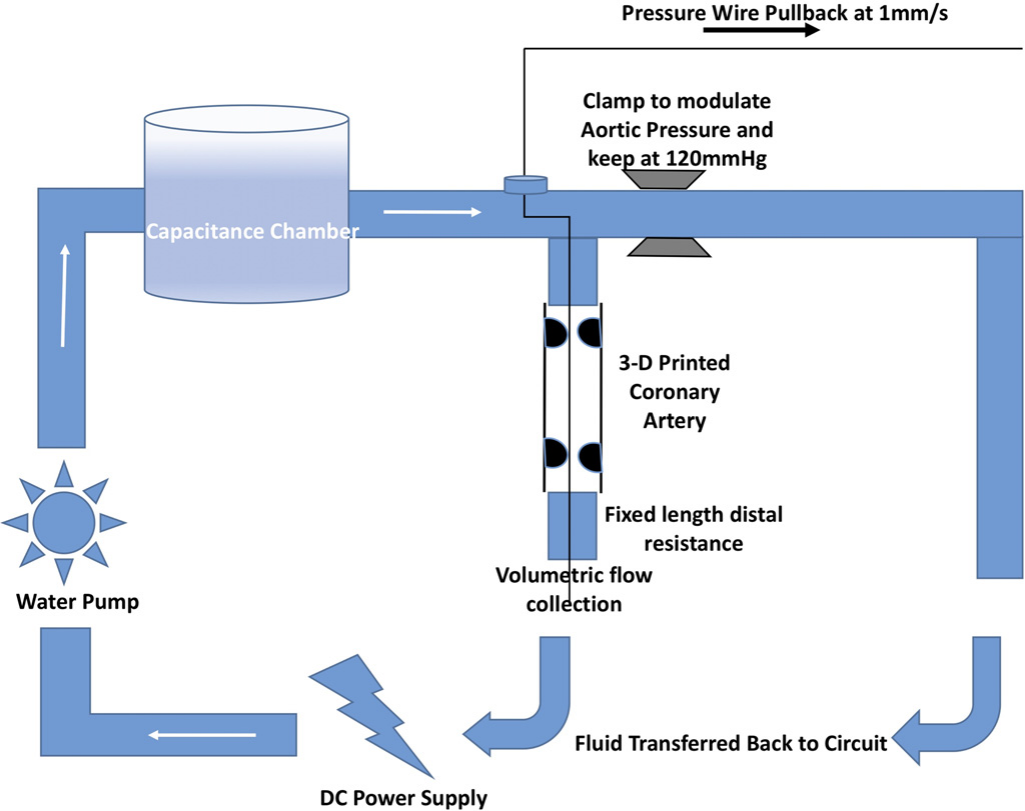
Diagrammatic representation of in vitro model of coronary circulation. Diagrammatic Representation of Continuous Flow Phantom model used to model serial stenosis hemodynamic interplay.

To model tandem disease, phantom tubes were created by 3D printing (Objet 500 Connex3™; 600×600×1600 dpi) from the biocompatible “TangoPlus FullCure PolyJet” photopolymer material (Objet Ltd, Israel) that has been shown to adequately model arterial compliance.^15^ The tubes created were 150 mm long, with an internal diameter of 5 mm and wall thickness of 2 mm to simulate the dimensions and compliance of a typical left anterior descending artery (Figure 1). A continuous flow model of coronary circulation was created to facilitate accurate measurement of pressure changes (Figure 2). Continuous inlet pressure was created by using an electrically driven water pump and capacitance chamber that connected to an aorta modeled from PolyJet tubing, with aortic pressure kept fixed. PolyJet tubing, modeling the left main coronary artery, then branched off the aorta with the 3D‐printed phantom attached, ensuring any connections themselves did not pose additional resistance (Figure 2). A fixed‐length low‐compliance silicon tubing was added downstream of the phantom tube and calibrated to create similar resistance to the coronary microcirculation during adenosine‐induced hyperemia. Distilled water was used as the fluid for all experiments, in line with previous in vitro models of coronary circulation^16^, ^17^ that presume Newtonian conditions are maintained within coronary arteries under physiological conditions.^18^ The use of 3D printing enabled all stenoses to have a fixed geometry to reduce variability in other factors influencing the pressure drop across a stenosis.^19^

The in vitro model was validated using the following tests (detailed in Figure S1): (1) A comparison of pressure measurements across a range of stenoses from our continuous‐flow model against measurements from a previously validated pulsatile flow model.^20^ (2) Triplicate pressure gradient measurements for each stenosis allowing calculation of intraclass correlation to ensure high test–retest reliability. (3) The compliance of 3D‐printed tubes was assessed by creating pressure–volume loops in a blank tube made of the same material, and compared with published data on coronary artery compliance.^15^ (4) The effect of luminal area reduction on the pressure drop across each stenosis was assessed and compared with previously reported coronary physiological data of pressure drops across stenoses in hyperemic conditions.^19^ (5) Volumetric flow rates measured at the end of the tandemly diseased phantom tubes were measured and plotted against pressure‐drops for each stenosis, enabling comparison with accepted pressure–flow relationships.^19^, ^21^

### Measurement of FFR

Using this setup, a pressure wire (Phillips Volcano™) was normalized to aortic pressure and advanced distal to the serial lesions. Total vessel FFR was calculated conventionally as the ratio of distal coronary pressure to aortic pressure, with conditions presumed equivalent to hyperemia given the continuous flow set‐up with fixed and minimal distal resistance. The pressure‐wire was pulled back through the lesions at 1 mm/s using a modified pullback device (Phillips Volcano R100) to generate FFR pullback curves with all experiments repeated in triplicate; mean values from these are quoted throughout (Figure 1). Each 3D‐printed configuration of tandem disease (varying in length of stenoses, separation, and severity) was then replaced with the corresponding 3D‐printed tube containing the isolated stenosis to simulate treatment with coronary angioplasty (PCI) (Figure 1).

For a given stenosis, the apparent trans‐stenotic pressure gradient (which in turn gives rise to ∆FFR_app_, assuming a fixed P_a_) is the change in pressure across a given lesion when the measurement was made in the presence of both lesions, whereas the actual trans‐stenotic pressure gradient (which gives rise to ∆FFR_true_, again assuming a fixed P_a_) is when that stenosis is present in isolation, with the accompanying stenosis removed (Figure 1). The extent of serial stenosis hemodynamic interplay was assessed by examining the magnitude of difference between ∆FFR_app_ and ∆FFR_true_, with various combinations of serial disease. The impact of the following on this difference was assessed: average severity (% diameter reduction) of both stenoses, length of stenoses (both physical length from 3D‐printed geometries and physiological length from pullback traces performed at 1 mm/s), physical separation distance between the serial stenoses, and the position of the stenosis in question (ie, proximal or distal to the accompanying stenosis).

### Generation of Equations to Predict FFR_true_ and Validation in a Clinical Cohort

Mathematical solutions to predict FFR_true_, the FFR in the vessel if the stenosis was present in isolation, were derived from the results within a derivation cohort of the in vitro study (see details in the Results section) and then tested within a small validation cohort of phantoms.

To demonstrate the clinical applicability of such a solution within real‐world cases of serial CAD, this solution was subsequently also tested in a clinical cohort of 30 patients with stable angina and coronary angiography that demonstrates serial disease within a main epicardial coronary artery (≥2 stenoses of >30% diameter reduction, at least 10 mm apart, with a plan to treat 1 lesion by PCI). All patients within the catheter laboratory cohort were loaded with 300 mg aspirin and 600 mg clopidogrel before being catheterized via the right radial artery using a standard 6F sheath with sedation, intra‐arterial nitrates, and weight‐adjusted heparin (70 μ/kg) given as standard. Following diagnostic angiography, an intracoronary pressure wire was inserted, calibrated, and manipulated to the distal vessel. A pullback of the pressure wire was subsequently performed during IV adenosine infusion (at a dose of 140 μg/kg per minute via an antecubital vein) before intervention, using the same 1 mm/s mechanical pullback method described for the in vitro study, with measurements made of the pressure gradient across each lesion in the presence of another (∆FFR_app_).

In the clinical setting, FFR_true_ was assessed following PCI of 1 of the 2 stenoses, with the treated lesion chosen by the operator using their standard clinical techniques. PCI was performed with 2nd/3rd‐generation drug‐eluting stents with optimization using intravascular imaging where appropriate.

The UK National Research Ethics Service approved the protocol in January 2016 (London Bridge Research Ethics Committee reference 15/LO/2011). All patients provided written informed consent before being enrolled in the study.

### Statistical Analysis

Data were analyzed using SPSS Statistics version 22 (released 2013; Armonk, NY: IBM Corp) and Graph Pad Prism Version 7.0a for Mac OS X. Data are expressed as mean±SD unless stated otherwise. Paired *t* tests were used to compare continuous variables after normality of data was visually assessed using histograms and Q‐Q plots. For all statistical analysis, *P*<0.05 is considered statistically significant. An empirical correction equation was developed using stepwise simple linear regression modeling. Details of the theoretical correction equation are stated in Figure S2. The incremental value of the correction equation compared with FFR_app_ from a conventional pressure‐wire pullback was determined by the proportion of cases where an incorrectly classified lesion (according to FFR threshold of 0.8) was correctly reclassified.

## Results

### In Vitro Model

Measurements of translesional pressure drop in the continuous‐flow model showed high test–retest reliability (intraclass correlation of 3 measurements for each lesion 0.97, *P*<0.05). The model demonstrated a nonlinear relationship between stenosis severity (quantified by diameter stenosis) and pressure gradient. In addition, the compliance and hysteresis loops generated by varying pressure and flow were similar to those reported in vivo.^22^ The model also showed strong statistical agreement (*r*=0.94, *P*<0.05) with measurements made in the previously validated pulsatile model.^20^ Furthermore, using volumetric flow‐rate measurements distal to the 3D‐printed phantoms, we were able to replicate accepted pressure drop versus flow velocity relationships.^19^ Validation data are summarized in Figure S1. In addition, as described in Figure 1, the concentric stenosis geometry meant that for severe lesions the phenomenon of “pressure recovery” was often observed because of the Bernoulli effect. Where any pressure recovery was observed (Figure 1), the pressure immediately proximal to the distal lesion, before pressure recovery phase, was taken as the interstenotic pressure to calculate ∆FFR_app_ and ∆FFR_true_.

**Figure 3:**
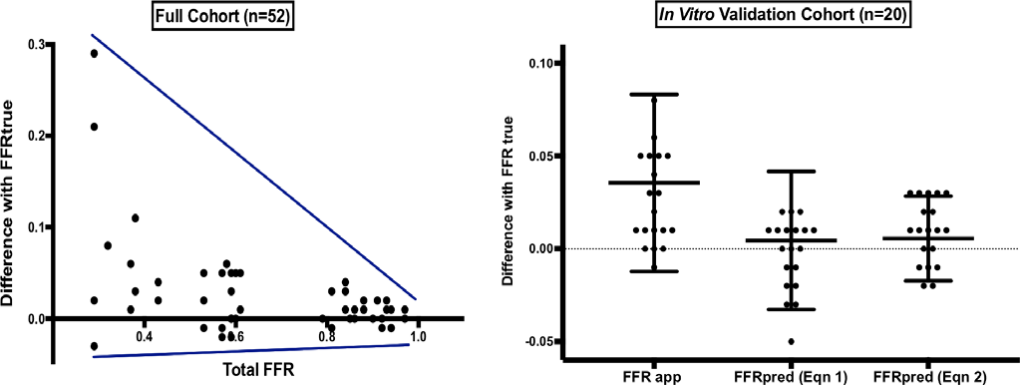
Results demonstrating the error in assessing pressure gradients in serial disease and how this can be corrected by applying correction equations generated from in vitro study. *Left:* In the full cohort of 52 tandemly diseased phantoms (with corresponding tube of stenoses in isolation), results demonstrated a significant (*P*<0.001) overestimation of FFR_true_ (and therefore an underestimation of stenosis significance): The more the data‐point is above the *x*‐axis, the greater the underestimation of a stenosis. The difference between FFR_app_ and FFR_true_ and the variance of this difference was greater with increasing burden of total disease in the vessel (lower total FFR). *Right:* In a randomly selected validation cohort of 20 phantoms, the error in estimating FFR_true_ was significantly reduced with the statistical regression equation (Eqn 1) with the variance and difference even further reduced using the theoretical correction equation (Eqn 2), based on knowledge of P_d_ and ∆P across the stenosis. FFR_app_ indicates apparent pressure gradient across a stenosis; FFR_pred_, predicted pressure gradient across a stenosis; FFR_true,_ true pressure gradient across a stenosis; P_d,_ distal coronary pressure.

In total, 52 combinations of tandem lesions were analyzed in vitro with average stenosis diameter stenosis of 55% and overall vessel FFR of 0.66±0.23. There was a significant difference between FFR_app_ and FFR_true_, with the true contribution of a given lesion being underestimated in the presence of an additional lesion in 85% of cases. The overall difference between the FFR_app_ and FFR_true_ was 17.1%, as a proportion of ∆FFR_true_ (absolute difference 0.036±0.048) with increasing lesion underestimation and variance between FFR_app_ and FFR_true_ with increasing cumulative burden of disease (represented by total FFR) (Figure 3). The degree of this underestimation was similar (*P*>0.05), regardless of whether the distal stenosis or proximal stenosis was removed.

The 3D‐printed model also enabled assessment of other factors on the extent of serial stenosis interplay including lesion length, distance between lesions, and physiological length. Results showed that none of these other factors correlated significantly with the extent of serial stenosis underestimation (*P*>0.05). Furthermore, we found no relationship between the finding of pressure‐recovery and the extent of serial stenosis interplay.

### Developing Algorithms to Predict FFR_true_

Our study showed total FFR and the magnitude of the pressure drop to be the strongest determinants of error in determining the physiological significance of each lesion. Based on these findings, 2 solutions were created to predict the true FFR of each stenosis: one empirically derived from regression modeling of the results from this study and the second from mathematical modeling of pressure drop and P_d_ from theoretical principles.

#### Empirical regression model

Based on the correlations observed in our study, multiple linear regression was performed to predict FFR_true_ (ie, residual FFR if the stenosis in question was present in isolation) from ∆FFR_app_ following a fixed‐rate pullback maneuver, using total vessel FFR as an input. This equation was created in a randomly selected derivation cohort of 32 and is summarized as follows:

$${\text{FFR}}_{\mathrm{predicted}}=0.9-\left(\Delta {\text{FFR}}_{\mathrm{app}}-\text{Total FFR}\times 0.11\right)$$

$${\text{FFR}}_{\mathrm{predicted}}=1-\frac{\Delta P}{P_{d}+\Delta P}$$

Although such a regression model is constrained by the parameters used in this experiment, we were able to perform proof‐of‐concept validation in the remaining 20 tandemly diseased tubes. We found the initial discrepancy versus ∆FFR_true_ was 17.1% (0.036±0.048) and improved significantly to 2.1% (0.005±0.04) with this equation, albeit still with a large degree of variance (Figure 3).

#### Theoretical model

The relationship between pressure and flow is curvilinear, but it has been established that most physiological measurements during hyperemia will fall within a relatively linear part of the curve. Our theoretical model of FFR estimation was therefore based on the assumptions of a linear pressure‐flow relationship. In this condition, the hemodynamic equivalent of Ohm's law is applicable, whereby the individual resistances of stenoses and the distal circulation stay fixed regardless of other stenoses being removed. Under these conditions, the theoretical FFR can be derived, without the need for a wedge pressure if we assume the variability of collateral flow across intermediate stenoses is minimal (see Figure S2 for full details):

In this equation, ΔP refers to the pressure drop across a lesion in the presence of serial disease and P_d_ refers to distal pressure. This relationship applies for both proximal and distal lesions and depends neither on the perfusion pressure (P_a_), nor the distal resistance R, and is solely a function of the measured pressure values. Using this equation, evaluation against the n=20 in vitro validation cohort yielded a markedly improved discrepancy of only 0.6% (0.006±0.02) against ∆FFR_true_ measurements (Figure 3). Given the more favorable results of this solution and the fact it was derived using first principles, not constrained by the model conditions from which it was derived, we then proceeded to test its performance in a clinical cohort of serial CAD.

### Testing Algorithms to Predict FFR_true_ in Clinical Cohort

**Figure 4:**
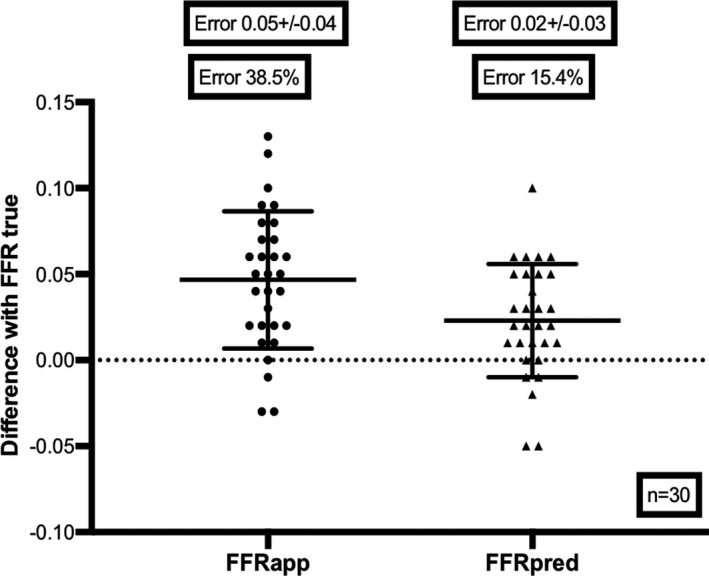
Predicting FFRtrue: clinical validation of correction equation. *Left:* Column scatterplot demonstrating the error in assessing FFR_true_ in the presence of accompanying disease (error 0.05±0.04; 38.5% as a proportion of true pressure gradient). *Right:* Column scatterplot demonstrating significant reduction in error in assessing FFR_true_ using our theoretically modeled correction equation (0.02±0.03, 15.4% as a proportion of true pressure gradient). FFR_app_ indicates apparent pressure gradient across a stenosis; FFR_pred_, predicted pressure gradient across a stenosis; FFR_true,_ true pressure gradient across a stenosis.

In the clinical cohort, mean total vessel FFR was 0.70±0.11 (see Table for further details of the vessels studied). These vessels contained tandem stenoses with mean diameter stenosis (by Quantitative Coronary Analysis) of 57.7±6.1%. Within this clinical cohort, the relative discrepancy of FFR_app_ and FFR_true_ was 38.5% (absolute difference 0.05±0.04), with no significant difference seen regardless of whether the proximal or distal lesion was being assessed. This improved significantly (*P*<0.001 by paired analysis) to 15.4% (absolute difference 0.02±0.03) with the theoretical correction equation (Figure 4). Importantly, in 4 out of 30 cases (13.3%), significant lesions were misclassified as nonsignificant using uncorrected FFR pullback alone (wrongly estimating that the residual FFR would have been ≥0.8 if the lesion was present in isolation). By applying the correction equation, lesions were misclassified in only 2 out of 30 cases (6.7%).

**Table 1:** Patient Demographic Data for Clinical Validation Cohort

Age, y	62±12
Male	27 (90)
Hypertension	18 (60)
Diabetes mellitus	10 (33)
Smoker	5 (17)
Hyperlipidemia	24 (80)
Tandemly diseased vessel	
LM—LAD	5 (17)
LAD	17 (57)
LCx	2 (7)
RCA	6 (20)
Lesion severity, QCA, %	57.7±6.1
Lesion length, QCA, mm	9.6±5.2
Distance between stenoses, mm	17.3±7.8

Values are n, n (%), or mean±SD. LAD indicates left anterior descending artery; LCx, left circumflex artery; LM, left main coronary artery; QCA, quantitative coronary angiography; RCA, right coronary artery.

Both within the in vitro and clinical validation cohorts, factors such as lesion length, lesion separation, and location of lesions did not significantly influence the performance of the equations in predicting FFR_true_. Unlike the in vitro cohort, we found that within the clinical validation cohort, significant pressure recovery was rarely found: a likely reflection of the fact that stenosis geometry is complex and nonconcentric. Where it was observed, as in the in vitro study, the straight line of pressure between lesions, before pressure recovery phase, was used to calculate FFR_app_ and FFR_true_.

## Discussion

The main findings of this study are as follows:

1. A physiologically representative model of serial CAD can be created in vitro using a continuous‐flow model based on 3D printing.
2. Within our model, we have demonstrated that stenosis underestimation commonly occurs when physiologically assessing serial CAD, regardless of whether a proximal stenosis is present alongside a more distal stenosis or vice versa. This underestimation is proportional to cumulative disease burden (the lower the total vessel FFR, the greater the likelihood of stenosis underestimation).
3. Using a theoretical solution derived from our in vitro experiments, we have created and validated an equation, derived from first principles, to predict to true functional significance of stenoses, using readily available data from routine pressure‐wire pullback measurements.
4. Within a clinical cohort, we have again shown significant underestimation of stenosis significance in serial CAD, with mean proportional error of 38.5% (0.05±0.04), which improved to 15.4% (0.02±0.03) using the prediction equation. Importantly, within this clinical cohort, 13.3% of all lesions studied were originally misclassified in the presence of serial disease, but with our mathematical solution, the misclassification rate more than halved, with only 6.7% of lesions misclassified.

The unique benefits of precisely controlling lesion geometry with 3D printing have enabled us to overcome many limitations of previous work in the field, where stenoses were created with external ties and clamps.^8^, ^17^ This has enabled us to show that the factors that most influence the error when predicting individual lesion significance in serial disease are total vessel FFR, average % diameter stenosis of the tandem stenoses, and pressure drop across the lesion in question. Other factors such as lesion separation and lesion length appear less important, with a statistically nonsignificant relationship with lesion underestimation. These findings are unsurprising because the % luminal narrowing caused by a stenosis contributes to both the linear and square coefficients within the equation that relates stenosis geometry to the pressure drop across it and as stated by Poiseuille's law, the resistance to flow is related to the 4th power of radius of the vessel.^23^

Compared with the in vitro cohort, in the clinical cohort we found greater residual error in estimating true physiological significance of a stenosis, despite the correction equation (15.4%). This is likely to reflect stenosis geometry variation that may alter the separation coefficient and turbulent conditions within a vessel. Furthermore, the lesions studied clinically showed greater variability in lesion length (Table), and although we have found this factor to not be as important in influencing serial stenosis underestimation, it is still likely to play a role, because it is an important contributor to both the frictional and separation coefficient of a stenosis. In addition, in the 3D‐printed in vitro setting, where we can replace a tandemly diseased phantom with the relevant isolated stenosis phantom, we get “perfect” revascularization with very little diffuse disease in the rest of the vessel. In the clinical setting, we know that this is more difficult to achieve with some degree of residual pressure gradient being common.^24^

Within the clinical cohort, the error because of serial stenoses of 0.05±0.04 may not initially seem significant at first glance but when compared with the mean change in FFR across the stenoses in question, we have shown it amounts to an error of >30%. Perhaps more significantly, current clinical guidelines recommend we use a single threshold to indicate whether we perform or defer revascularization (for FFR the contemporary threshold now stands at 0.8).^6^ When applying these rigid thresholds to the individual lesions without our clinical data set, we found 13.3% of lesions were misclassified (using an FFR threshold of significance as <0.8). With the use of our equation, this rate of misclassification halved to 6.7%. If these were cases of serial stenoses involving the left main coronary artery, this could potentially mean we can more than halve the rate of inappropriate revascularization strategies being chosen on the basis of physiology.

Our study represents a significant advance in evaluating tandem stenoses. For the first time we have been able to objectively evaluate the nature of serial stenosis interplay using novel 3D printing of CAD. We have subsequently generated a correction equation, not requiring the measurement of wedge pressure between lesions, that is solely based on routine pressure‐wire pullback measurements and shown a significant improvement in the prediction of the true physiological significance of lesions within a clinical cohort of 53 patients. The only previous study with clinical validation of an in vitro solution to use with FFR was in a smaller cohort of 32 patients with results similar to our own but with the requirement of measuring wedge pressure between stenoses.^8^, ^11^ Importantly, our solution does not need wedge pressure measurements and so requires no ballooning or the need to mandate starting PCI before the optimum revascularization strategy is chosen. In Figure S3, we describe an example clinical case of serial CAD that demonstrates the utility of the mathematical correction model we have described.

In this study, our goal was to derive a simple (even if imperfect) solution based on information that is readily and routinely available in the catheter laboratory. Extracting accurate geometric information from a standard coronary angiogram is difficult, with quantitative coronary analysis seeming both retrograde and inadequate. However, when lesion characteristics (which can be accurately obtained in the 3D‐printed phantom) were incorporated, the predictive accuracy of the mathematical solution was even better than with intracoronary pressure–based inputs alone, suggesting even more improvement is possible if in vivo 3D stenosis geometry were to be characterized better. Future directions will involve applying some of the findings of our work to computed tomography and FFR_CT_ outputs in patients who have had prior computed tomography imaging, because the latter lends itself to improved geometric characterization.

In summary, this study has developed a mathematical correction model that significantly improves the prediction of true stenosis significance in serial CAD. The next steps will be to carry out a multicenter clinical study to test the clinical utility of our corrected FFR pullback solution and also to compare it with other conventional techniques, including resting indices such as iFR pullback.^14^

### Limitations

Tandem stenoses within humans are rarely found in otherwise smooth vessels, as has been modeled within our 3D‐printed phantoms. The reality is that between stenoses, diffuse atheroma acts as a continuous mild stenosis and this is likely to represent one of the reasons why the equation was shown to perform less well in the clinical cohort, albeit still resulting in a significant improvement to current methods.

An ideal model for predicting FFR_true_ would be based on the varying curvilinear relationships between pressure and flow but this would not be possible from pressure measurements alone. We acknowledge this may be one of the reasons there is still some residual error when using a correction equation that is based on a linear pressure‐flow relationship in physiological conditions. By doing so, however, we have been able to establish a simple model utilizing pressure‐wire data, without the need for simultaneous flow or wedge pressure measurement, which represents a significant improvement to current methods as evident in the clinical and phantom validation cohorts.

The use of resting physiological indices, particular iFR, has been growing for the assessment of serial disease, with proponents suggesting that without hyperemia, there is less serial stenosis hemodynamic interplay. Before universal adoption, the FFR correction model would require an assessment of its performance against other physiological indices commonly used in serial disease, such as iFR.

## Conclusions

This study demonstrates that estimation of the true stenosis significance in serial disease is prone to significant underestimation using FFR, regardless of whether is there is accompanying distal or proximal disease. 3D‐printed modeling of tandem disease has enabled us to generate and test a mathematical equation to improve estimation of the true physiological impact of each stenosis, using readily available measurements from a routine pressure‐wire study. Within a clinical validation cohort, using measurements after isolation of the stenosis following PCI, we have shown that incorporating this equation into conventional FFR assessments can significantly improve the prediction of the true lesion significance, with a large reduction in stenosis misclassification.

## Sources of Funding

Modi and Ryan are funded by British Heart Foundation Clinical Research Training Fellowships (FS/15/78/31678 and FS/18/16/33396, respectively).

## Disclosures

None.

## Supporting information


**Figure S1.** Validation of in vitro model of coronary circulation.
**Figure S2.** Theoretical model derivation.
**Figure S3.** Example clinical case demonstrating utility of mathematical correction model.
