# Supplementary material for: Optimal Application of Fractional Flow Reserve to Assess Serial Coronary Artery Disease: A 3D‐Printed Experimental Study With Clinical Validation
Source: J Am Heart Assoc. 2018 Oct 14;7(20):e010279. doi: 10.1161/JAHA.118.010279 (PMC6474982; doi:10.1161/JAHA.118.010279)
Supplement: Supplementary file 1 — Figure S1. Validation of in vitro model of coronary circulation. Figure S2. Theoretical model derivation. Figure S3. Example clinical case demonstrating utility of mathematical correction model. [file JAH3-7-e010279-s001.pdf]

SUPPLEMENTAL MATERIAL

Figure S1. Validation of *in vitro* Model of Coronary Circulation.

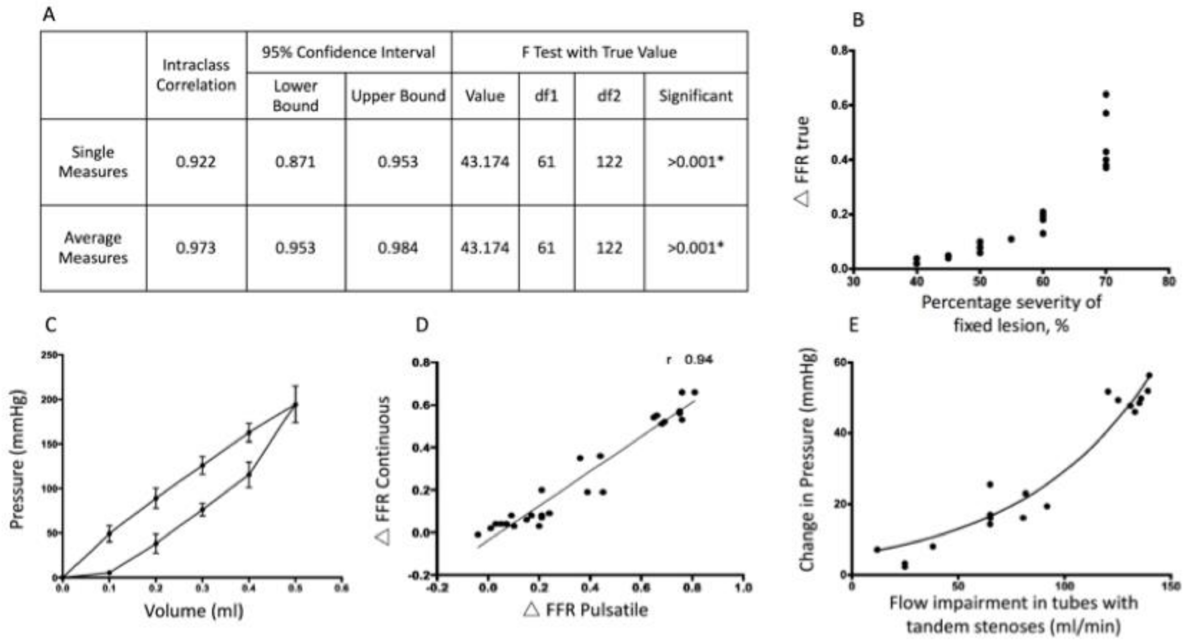

	Intraclass Correlation	95% Confidence Interval		F Test with True Value			
		Lower Bound	Upper Bound	Value	df1	df2	Significant
Single Measures	0.922	0.871	0.953	43.174	61	122	>0.001*
Average Measures	0.973	0.953	0.984	43.174	61	122	>0.001*

Summary of Results from Model Validation Experiments. A: Intra-class Correlation Table for both individual and mean measurements of Delta FFR. B: Scatterplot showing the effect of lesion severity (% diameter reduction) on delta FFR. C: Pressure-volume loop acquired in the compliance testing. Data represent mean ± standard deviation. D: Continuous vs. Pulsatile flow system Spearman correlation analysis test. E: Scatterplot demonstrating the quadratic relationship between volumetric flow velocity impairment (versus blank tube with no stenoses) and the pressure drop within the tandemly diseased tubes

Figure S2. Theoretical Model Derivation.

Consider the pressure and resistance distribution in a vessel with tandem lesions pre and post-PCI (Figure below). For illustration it is assumed that the distal lesion (R_2) is treated in this derivation, however, the outcome is identical if the proximal lesion is removed instead.

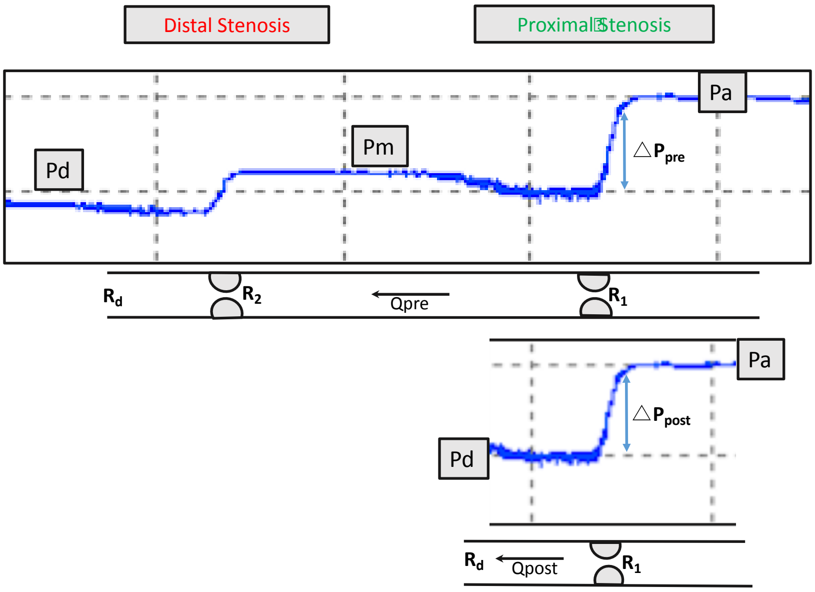

Since it is assumed that the resistance of the untreated lesion remains unchanged (i.e. R_1 and R_2 are independent) and flow remains constant throughout the vessel (i.e. no side-braches between the lesions), Ohm's law applied to pre- and post-PCI setting leads to:

$$Q_{pre} = \frac{\Delta P_{pre}}{R_1} \quad \text{and} \quad Q_{post} = \frac{\Delta P_{post}}{R_1}$$

which can be combined to give $$\frac{Q_{post}}{Q_{pre}} = \frac{\Delta P_{post}}{\Delta P_{pre}} \qquad (1)$$

In addition, similar expressions can be written for the distal resistance R_d (which is assumed to be fixed):

$$Q_{pre} = \frac{P_d}{R_d}$$

$$Q_{post} = \frac{P_a - \Delta P_{post}}{R_d}$$

which combine to give

$$\frac{Q_{post}}{Q_{pre}} = \frac{P_a - \Delta P_{post}}{P_d} \qquad (2)$$

Equations (1) and (2) can be used together to eliminate flows

$$\frac{\Delta P_{post}}{\Delta P_{pre}} = \frac{P_a - \Delta P_{post}}{P_d}$$

Rearranging for ΔP_{post} yields the following expression

$$\Delta P_{post} = \frac{\Delta P_{pre} P_a}{P_d + \Delta P_{pre}} \tag{3}$$

Now, using the definition of post-PCI FFR

$$FFR_{model} = \frac{P_a - \Delta P_{post}}{P_a} = 1 - \frac{\Delta P_{post}}{P_a} = 1 - \frac{\Delta P_{pre}}{P_d + \Delta P_{pre}}$$

where equation (3) has been used in the last step.

Figure S3. Example Clinical Case Demonstrating Utility of Mathematical Correction Model.

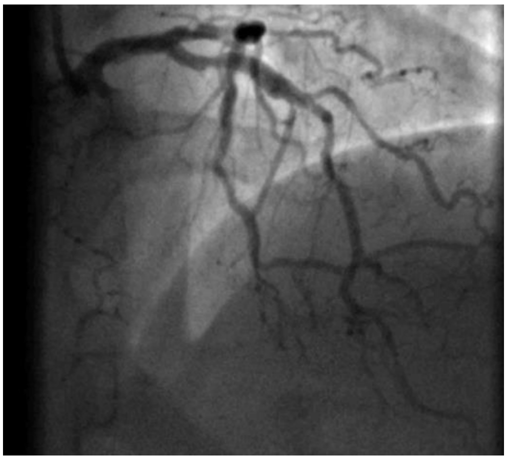

LAD demonstrating serial lesions.

Question about what the residual FFR would be due to distal lesion once proximal LAD lesion treated

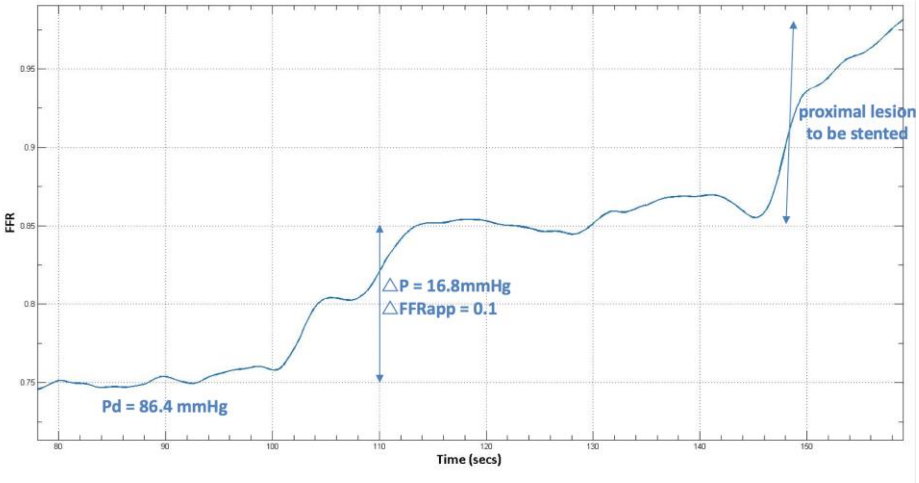

distal FFR of 0.75

(distal Pd of 86.4mmHg)

hyperemic pressure-wire pullback demonstrating apparent ΔP and apparent ΔFFR

without model, it would be estimated that residual FFR from distal stenosis only would be 0.90

$$\boldsymbol{FFR_{model} = 1 - \frac{\Delta P_{pre}}{P_d + \Delta P_{pre}} = 1 - \frac{16.8}{103.2} = 0.84}$$

with equation, residual FFR following PCI of proximal lesion estimated to be 0.84
